# Verona Integron-Encoded Metallo-Beta-Lactamase (VIM)–Producing *Pseudomonas aeruginosa* Outbreak Associated with Acute Care

**DOI:** 10.1017/ash.2021.47

**Published:** 2021-07-29

**Authors:** Allison Chan, Alicia Shugart, Albert Burks, Christina Moore, Paige Gable, Heather Moulton-Meissner, Gillian McAllister, Alison Halpin, Maroya Walters, Amelia Keaton, Kelley Tobey, Katie Thure, Sarah Schmedes, Paige Gable, Henrietta Hardin, Adrian Lawsin

## Abstract

**Background**: Contaminated healthcare facility plumbing is increasingly recognized as a source of carbapenemase-producing organisms (CPOs). In August 2019, the Tennessee State Public Health Laboratory identified Tennessee’s twelfth VIM-producing carbapenem-resistant *Pseudomonas aeruginosa* (VIM-CRPA), from a patient in a long-term acute-care hospital. To determine a potential reservoir, the Tennessee Department of Health (TDH) reviewed healthcare exposures for all cases. Four cases (33%), including the most recent case and earliest from March 2018, had a history of admission to intensive care unit (ICU) room X at acute-care hospital A (ACH A), but the specimens were collected at other facilities. The Public Health Laboratory collaborated with ACH A to assess exposures, perform environmental sampling, and implement control measures. **Methods**: TDH conducted in-person infection prevention assessments with ACH A, including a review of the water management program. Initial recommendations included placing all patients admitted to room X on contact precautions, screening for CPO on room discharge, daily sink basin and counter cleaning, and other sink hygiene measures. TDH collected environmental and water samples from 5 ICU sinks (ie, the handwashing and bathroom sinks in room X and neighboring room Y [control] and 1 hallway sink) and assessed the presence of VIM-CRPA. Moreover, 5 patients and 4 environmental VIM-CRPA underwent whole-genome sequencing (WGS). **Results:** From February to June 2020, of 21 patients admitted to room X, 9 (43%) underwent discharge screening and 4 (44%) were colonized with VIM-CRPA. Average room X length of stay was longer for colonized patients (11.3 vs 4.8 days). Drain swabs from room X’s bathroom and handwashing sinks grew VIM-CRPA; VIM-CRPA was not detected in tap water or other swab samples. VIM-CRPA from the environment and patients were sequence type 253 and varied by 0–13 single-nucleotide variants. ACH A replaced room X’s sinks and external plumbing in July. Discharge screening and contact precautions for all patients were discontinued in November, 5 months following the last case and 12 consecutive negative patient discharge screens. Improved sink hygiene and mechanism testing for CRPA from clinical cultures continued, with no new cases identified. **Conclusions:** An ICU room with a persistently contaminated sink drain was a persistent reservoir of VIM-CRPA. The room X attack rate was high, with VIM-CRPA acquisition occurring in >40% of patients screened. The use of contaminated plumbing fixtures in ACH have the potential to facilitate transmission to patients but may be challenging to identify and remediate. All healthcare facilities should follow sink hygiene best practices.

**Funding:** No

**Disclosures:** None

